# Microwave Enabled Physically Cross Linked Sodium Alginate and Pectin Film and Their Application in Combination with Modified Chitosan-Curcumin Nanoparticles. A Novel Strategy for 2nd Degree Burns Wound Healing in Animals

**DOI:** 10.3390/polym13162716

**Published:** 2021-08-13

**Authors:** Hafiz Muhammad Basit, Muhammad Ali, Mian Mufarih Shah, Shefaat Ullah Shah, Abdul Wahab, Hassan A. Albarqi, Abdulsalam A. Alqahtani, Ismail A. Walbi, Nauman Rahim Khan

**Affiliations:** 1 Department of Pharmaceutics, Faculty of Pharmacy, Gomal University, DIKhan 29050, Pakistan; basitkhan053@gmail.com (H.M.B.); alipharm33@gmail.com (M.A.); shefaatbu@gmail.com (S.U.S.); 2 Gomal Centre for Skin/Regenerative Medicine and Drug Delivery Research, Faculty of Pharmacy, Gomal University, DIKhan 29050, Pakistan; 3 Department of Medicine MTI, Hayatabad Medical Complex, Peshawar 25000, Pakistan; mianmufarih458@gmail.com; 4 Department of Pharmacy, Kohat University of Science and Technology, Kohat 26000, Pakistan; wahabscholar@yahoo.com; 5 Department of Pharmaceutics, College of Pharmacy, Najran University, Najran 55461, Saudi Arabia; haalbarqi@nu.edu.sa (H.A.A.); aamari@nu.edu.sa (A.A.A.); 6 Department of Clinical Pharmacy, College of Pharmacy, Najran University, Najran 55461, Saudi Arabia; iawalbi@nu.edu.sa

**Keywords:** burn wound healing, polymeric film, microwave, nanoparticles-film combined application

## Abstract

This study reports microwave assisted physically cross-linked sodium alginate and pectin film and their testing in combination with modified chitosan-curcumin nanoparticles for skin tissue regeneration following 2nd degree burn wound. Film was formulated by solution casting method and physically cross-linked using microwave irradiation at frequency of 2450 MHz, power 750 Watt for different time intervals for optimization. The optimized formulation was analyzed for various physiochemical attributes. Afterwards, the optimized film and optimized modified chitosan-curcumin nanoparticles were tested in combination for skin regeneration potential following burn wound in vivo and skin samples extracted and tested for different attributes. The results indicated that the optimized film formulation (5 min microwave treatment) physicochemical attributes significantly enhanced addressing the properties required of a wound healing platform. The vibrational analysis indicated that the optimized film experienced significant rigidification of hydrophilic domains while the hydrophobic domains underwent significant fluidization which also resulted in significant increase in the transition temperatures and system enthalpies of both polymer moieties with microwave treatment. The combined film and nanoparticles application significantly increased protein content in the wounds which were evident from higher absorbance ratios of amide-I and amide-II (2.15 ± 0.001), significantly higher melting transition temperature and enthalpy (∆T = 167.2 ± 15.4 °C, ∆H = 510.7 ± 20.1 J/g) and higher tensile strength (14.65 ± 0.8 MPa) with significantly enhanced percent re-epithelization (99.9934 ± 2.56) in comparison to other treatments. The combined application of film and nanoparticles may prove to be a new novel treatment strategy for 2nd degree burn wound healing.

## 1. Introduction

As the largest human organ, skin is often prone to easy damage thus compromising the barrier properties and enabling exogenous pathogens to infiltrate the wound and complicating the wound as well as delaying the wound healing process [1]. Burn wound treatment is still a challenge for biomedical scientists since they also leave behind scars which drastically compromise the psychological wellbeing of the patient. As per WHO statistics, a total of 11 million burn cases happen globally each year, of which 180,000 cases are fatal [2]. The skin tissue engineering is a prospective source of advanced therapy which is extensively studied these days to combat acute and chronic skin injuries [3]. It involves development of platforms with ability to either replace the entire tissue and/or help in creating suitable cell environment which help in restoring the normal function of the damaged tissue [4]. Some of the epidermal [5], dermal [6] and epidermal/dermal composite [7] skin substitute had already been marketed, but their high cost is the major hindrance for patients from a developing country like Pakistan. Thus, it is always imperative to look for an alternative treatment protocol to treat burn injuries.

Polymeric films have long been utilized as a wound healing platform for skin tissue regeneration applications [8] and various polymers has thus been used for this purpose [9,10,11,12,13]. Films are not only easy to prepare, but also their physicochemical properties can easily be modified to address the properties required of an ideal wound healing platform [14,15,16,17].

Sodium alginate and pectin are two natural polysaccharide polymers and due to their inherent biocompatibility, biodegradability, nontoxicity and non-immunogenicity, serve as an ideal candidates for fabricating wound healing platforms [18].They have long been employed in fabricating controlled drug delivery systems [19,20], for cellular encapsulation [21], as an immune stimulatory agent [22], in cosmetics [23], as scaffold in tissue engineering [24], wound dressing material [25], fabrication of bandages [26], as a disintegrating agent in tablets [27], bone regeneration scaffolds [24], as a dental impression material [28] and soft tissue engineering [29], for enhanced absorption of wound exudates [30], and as a protective barrier against bacterial infection [31]. For synergistic wound healing effect, alginate and pectin had also been employed as a blend with good physicochemical properties [32], were found to be noncytotoxic [33] and promote skin regeneration [34]. The simple mixing of polymers is often not enough to address the demerits associated with lone use of either polymer, thus cross linking using chemical cross linkers is often employed [32,35].

Physical cross linking using microwave represents a feasible cross-linking technique which is devoid of demerits associated with use of chemical cross linkers [36]. Microwave, an electromagnetic wave with frequencies in the range of 300 MHz to 300 GHz has numerous biomedical [37,38,39,40,41,42,43] and pharmaceutical applications [44,45,46,47,48,49,50,51], as well as applications in food processing [52]. Keeping in view the ability of microwave to affect the macromolecular rearrangement via hydrogen bonding, electrostatic interaction and/or hydrophobic interactions, it is envisaged that the physically cross-linked sodium alginate and pectin blend films using microwave may enhance their physicochemical properties thus making them best suited for wound healing applications. Thus, the objective of this study was to investigate the microwave enabled physically cross-linked polymer blend films alone and in combination with modified-chitosan curcumin nanoparticles to facilitate skin tissue regeneration following 2nd degree burn wound in animals. The modified-chitosan curcumin nanoparticles portion of our project is published earlier [53].

## 2. Materials and Methods

### 2.1. Chemicals

Sodium alginate (Sigma Aldrich, St. Louis, MO, USA) and pectin (Sigma Aldrich, St. Louis, MO, USA) were used for film preparation. Monobasic potassium phosphate (Sigma Aldrich, St. Louis, MO, USA), disodium hydrogen orthophosphate (Merck, Darmstadt, Germany), hydrochloric acid (Merck, Darmstadt, Germany), Tween80 and Glycerol were graciously supplied by Biolabs, Islamabad, Pakistan. All chemicals were used without any further purification.

### 2.2. Film Formulation

The film formulations were prepared using solution casting method. Briefly, an accurately weighed 2 g each of sodium alginate and pectin were dispersed separately in 93.9 g distilled water under continuous magnetic stirring. Following complete dissolution, 0.1% tween 80 and 2% glycerol were added and mixed thoroughly. Both the polymer solutions were mixed in another beaker in ratio of 1:1 and again stirred thoroughly till complete homogenization. Accurately weighed 50 g bubble free polymer mixture solution was poured into a glass petri dishes (diameter 8.5 cm, area 56.75 cm^2^) and placed in an oven (DO-100NG, SH Scientific, Daejeon, Korea) at 40 ± 2 °C till complete drying. When required, the polymer mixture solution in a petri dish was subjected to microwave treatment at frequency of 2450 MHz at power of 750 Watt with varying treatment time for the purpose of optimization followed by incubation in oven for drying. The dried films were peeled off from the petri dishes, cut into specific dimensions (25 mm diameter) and stored in desiccator till subjection to physicochemical characterization tests described below. The formulations are given in Table 1.

### 2.3. Moisture Adsorption

The moisture adsorption test was performed using an already reported method [54]. Briefly, the dried film samples with known dimensions (25 mm) were placed in a desiccator prefilled with calcium sulphate and incubated for 48 h (at RH of 0%), then their dried weight (*W*) was determined. Subsequently, the same samples were then transferred into another desiccator prefilled with saturated solution of potassium sulphate and incubated again for the next 48 h (at RH of 97%) enabling them to maximally absorb moisture. The samples were removed and weighed again individually to determine their moist weight (*W*0). The percent moisture adsorption was then calculated using the following relation [13].

(1) $$MA\;\left(\%\right)=\frac{W0-W}{W}\times 100$$

The experiment was repeated at least three times for each film and results averaged.

### 2.4. Water Vapor Transmission (WVTR) and Vapor Permeability Rate (WVP)

The *WVTR* and *WVP* of the films were determined using the ASTM method (ASTM E96-93, 1993) with some modifications. Briefly, the film samples were cut into dimensions of 33 mm and tightly placed onto the neck of glass bottle prefilled with saturated solution of potassium sulphate for providing virtual relative humidity of 97%. The bottles were placed in a desiccator containing anhydrous calcium sulphate to maintain relative humidity of 0%. The desiccator containing the bottle was then transferred into an oven maintained at 25 ± 1 °C. Each bottle was individually weighed at time interval of 1 h for total of 12 h and the water vapor transmission rate across the film was calculated from the weight loss of the bottle. The average weight change as a function of time was plotted and slope determined from the linear regression of the curve. The experiment was repeated six times and results averaged. The *WVTR* and *WVP* were estimated from following equations [13].

(2) $$WVTR=\frac{Slope}{Film\;area}$$

where slope is the slope of the graph of calculated from the weight loss vs. time curve and film area was 0.000903 m^2^.

(3) $$WVP=\frac{WVTR\times T}{∆P}$$

where *T* is the mean film thickness (mm); Δ*P* is the partial water vapor pressure difference (mmHg) across the two sides of the film specimen (the partial vapor pressure of water at 25 °C = 23.73 mmHg).

### 2.5. Erosion and Water Uptake

An accurately weighed (initial weight *Wi*) and known dimension (25 mm) of the film samples were placed in a petri-dish containing 20 mL phosphate buffer pH 7.4 simulating the pH of the wound bed conditions and incubated in an oven (BOV 50V, Biobase, Jinan, China) at 37 °C ± 0.2 °C for a duration of 30 min. At specific time intervals, film samples were removed from buffer solution and blot dried by gently sliding them on the surface of the petri dish to remove adhered moisture and weighed (*Wt*). Afterwards, the same film samples were placed in an oven at 40 ± 0.2 °C for at least 5 days. The dried films were then transferred to a desiccator maintained at 25 °C, enabling the films to achieve a constant weight and weighed again (*Wd*). The percent erosion (*E*) and percent water uptake (*WU*) were calculated using the following equation [13].

(4) $$E\;\left(\%\right)=\frac{Wi-Wt}{Wi}\times 100$$

(5) $$WU\;\left(\%\right)=\frac{Wt-Wd}{Wd}\times 100$$

The test was performed for each sample in triplicate and results averaged.

### 2.6. Tensile Strength

The mechanical strength of films was determined under room conditions of temperature and relative humidity using Universal testing machine (Testometrics, Rochdale, UK). The film samples were cut into strips, 7.5 cm in length and 3.5 cm in width, and fixed between the jaws of the machine. The initial grip separation and cross-head speed were set to 50 mm and 5 mm/min, respectively. Sample was pulled with 50 N loads. The maximum force to break the film was recorded. The experiment was repeated three times and results averaged.

### 2.7. Vibrational Spectroscopic Analysis

The characteristics peaks of the dried polymeric films were recorded by an ATR-FTIR spectrophotometer (UATR TWO, Perkin Elmer, Buckinghamshire, UK). Each film and/or powdered polymer was placed on the surface of the diamond crystal and clamped to ensure close contact and high sensitivity. All the samples were scanned over wave number range of 400 to 4000 cm^−1^ with an acquisition time of 2 min. Each sample was analyzed three times and results averaged.

### 2.8. Thermal Analysis

The changes in the transition temperature of the polymeric film were recorded via differential scanning calorimetry (PerkinElmer Thermal Analysis, Waltham, MA, USA). An accurately weighed 4 to 6 mg of the polymeric film was sealed in standard aluminum pan and heated from 0 to 300 °C under continuous flow of nitrogen gas at rate of 40 mL/min. The characteristic peak temperature and enthalpy of the system were recorded. Each sample was analyzed three times and results averaged.

### 2.9. Morphology

Surface morphology of the films was analyzed using ultra-high resolution field emission scanning electron microscope (UHR-FESEM, MERLINN/344999-9001-030, Zeiss, Germany). The films were mounted onto stubs (12 mm diameter) using double-sided adhesive carbon tape. The films were then sputter coated with gold for 5 min (QUORUM Sputter Coater Q150R S, Quorum, UK) before placing in the chamber of the microscope and images acquired using SmartTiff software. Images were taken at magnification power of 100×, 500×, 1000×, 2000× and 3000×.

### 2.10. In Vivo Wound Healing

Healthy male Sprague Dawley rats (age of 3 months, weighing 220–250 g) were used for in vivo wound healing studies. For this purpose, rats were acclimatized for 7 days with free access to food and water under temperature conditions of 25 ± 2 °C and relative humidity at 65 ± 5%. Prior to wound infliction, all animals were kept on fasting for 24 h and were then randomly divided into four groups: untreated group, film, nanoparticles and film-nanoparticles combined group with a total of 8 animals per group. All animals were anesthetized by intra-muscular injection of ketamin (100 mg/kg) and xylazine (10 mg/kg) mixture followed by shaving of the back hairs with sharp blade, cleansed by ethanol swab and area marked followed by infliction of 2nd degree burn wound. An already reported method was used for 2nd degree burn wound infliction [55]. Briefly, contact partial thickness wound was inflicted in each animal on shaved dorsal region using deionized water preheated to 65 ± 5 °C through adhering a plastic tube (internal diameter 14.4 mm, external diameter 16.5 mm) on shaved region using UHU gum followed by addition of 6 mL of preheated water into it and kept for 1 min in 9 repetitive cycles. Following wound infliction, the formulations were applied onto the wounded area with the aid of standard gauze and 3 M adhesive tape. The untreated animal group received only standard gauze application. The treatment group (nanoparticles, film, film-nanoparticles combined) received either 120 mg nanoparticles application alone, or optimized film application alone or nanoparticles application followed by covering the wounded area with optimized film. The treatments were applied on daily basis and dressing changed accordingly till complete healing of the wound. When required, animals were sacrificed by cervical dislocation on day 14 and skin containing the wound surgically excised, washed with normal saline and stored at −20 °C till further use. All animal experimentation was conducted in accordance with the institutional ethics regulations adapting the international guidelines (OECD Environment, Health and Safety). The study protocol was approved by the institutional ethical review board vide reference No. 503/QEC/GU of the Gomal University Pakistan.

### 2.11. Wound Morphology

The surface morphology of the wound was recorded using a digital camera (Canon D5100, Tokyo, Japan) from a fixed distance and angle in the absence of formulation and/or gauze at intervals of day 0, day 2, day 7, day 10, day 14 and day 18. The size of the wound was determined by analyzing the photographs using Image J software (version.1.53h) and the percent re-epithelization determined using the following equation.

(6) $$Reepithelization\;\left(\%\right)=\frac{\left(Wound\;size\;at\;t=0\right)\;-\;\left(Wound\;size\;at\;t\right)}{Wound\;size\;at\;t=0}\times 100$$

where the wound size was an average measurement from the longest and shortest dimensions of the wounded area.

### 2.12. Skin Histology

The newly regenerated skin tissue containing the wound was subjected to histological examination. For this purpose, the stored skin samples were thawed at room temperature for 3 h followed by fixation in 10% formalin aqueous solution for 3 days under ambient conditions and then preceded by trimming of skin samples, washed with normal saline and dehydrated in ethanol. The dehydrated skin samples were cleansed using xylene and embedded in paraffin wax. Sections of 5 µm thickness were prepared using a microtome (HM-340E, Microm Inc., Boise, ID, USA) and subjected to H&E (hematoxylin and eosin) and Masson’s trichrome separately. The slides were viewed, and respective sections photographed using an inverted microscope fitted with a camera (HDCCE—X5N).

### 2.13. Vibrational and Thermal Analysis of Skin

The vibrational spectra of dermal layer of the untreated as well as treated animal groups skin samples were recorded at a resolution of 16 cm^−1^ over a wave number range of 400 to 4000 cm^−1^ with an acquisition time of 2 min using ATR-FTIR (UATR TWO, Perkin Elmer, Buckinghamshire, UK). The corresponding ATR-FTIR spectra were compared to estimate extent of collagen deposition. For this purpose, the corresponding absorbance of amide-I and amide-II were recorded which originates from the protein contents of the skin. The ratio of absorbance of treated group to control group was used as a novel technique to estimate the extent of collagen and/or protein deposition. Each sample was analyzed three time and results averaged.

The skin samples containing wound were also subjected to thermal analysis using DSC, (Perkin Elmer, Thermal Analysis, Boston, MA, USA) for the purpose of estimating changes induced in the lipidic and proteinous domains of skin with various treatments applied in comparison to control skin samples. Briefly, an accurately weighed 3 mg of the full thickness skin containing wound was carefully trimmed and sealed in standard aluminum pan followed by subjection to thermal analysis in temperature range of 30 to 180 °C at a heating rate of 10 °C/min under continuous pulse of nitrogen gas at flow rate of 40 mL/min. The melting temperature (∆T) and enthalpy (∆H) corresponding to lipidic and proteinous domains were recorded. Each sample was analyzed at least three times and results averaged.

### 2.14. Tensile Strength of Skin Samples

The excised skin samples were trimmed into strips of 5 cm length and 2.5 cm width and subjected to tensile strength measurement (Testometric M-500, Rochdale, UK). The strips were affixed between the upper and lower jaw of the tensiometer and uniaxially pulled at test speed of 5 mm/s and withdrawal speed of 10 mm/s respectively using load of 30 kg. The maximum force required to break the skin samples and break point was recorded. Each skin sample was examined three times and results averaged.

### 2.15. Statistical Analysis

All values are expressed as a mean of three readings with respective standard deviation. Student’s *t*-test and/or analysis of variance (ANOVA) followed by post-hoc analysis was used with the level of significance set at *p* < 0.05.

## 3. Results

### 3.1. Moisture Adsorption

**Figure 1:**
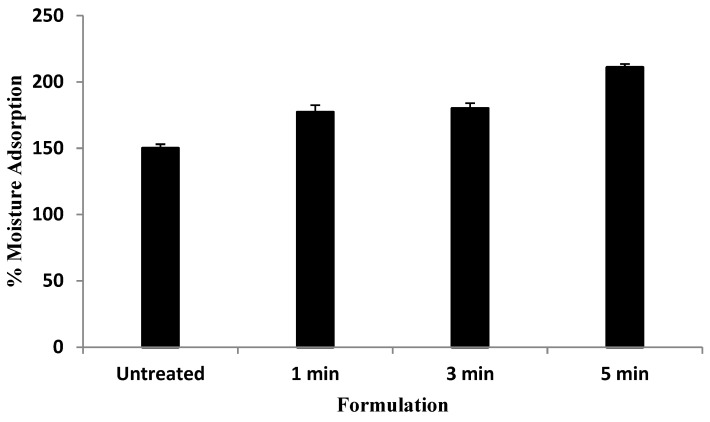
Percent moisture adsorption of various blend films.

The efficient moisture adsorption ability of film helps remove wound exudates from the wound bed which not only helps in wound debridement but also keeps the wound moist which is detrimental for fast skin regeneration [56]. Furthermore, a wet microenvironment also ensures fewer aberrations and the least scar formation [57,58]. The moisture adsorption results of untreated and microwave-treated sodium alginate and pectin blended films are shown in Figure 1. The moisture adsorption capacity of film is directly related to microwave treatment time, where significantly higher moisture (Student’s *t*-test, *p* < 0.05) adsorption was observed with blend treated for 5 min in comparison to untreated as well as 1 min and 3 min treated film, respectively.

### 3.2. Water Vapor Transmission Rate (WVTR) and Water Vapor Permeability (WVP)

The water vapor transmission and water vapor permeability potential are important physical characteristics of a polymeric film where reduced water loss from the wound bed into the external environment is of pivotal importance in facilitating the wound healing process [59]. The water vapor transmission and water vapor permeability results are shown in Table 2. It is evident from the results that the microwave treatment induced a significant increase (Student’s *t*-test, *p* < 0.05) in the *WVTR* and *WVP* in comparison to untreated blend film.

### 3.3. Erosion and Water Uptake

**Figure 2:**
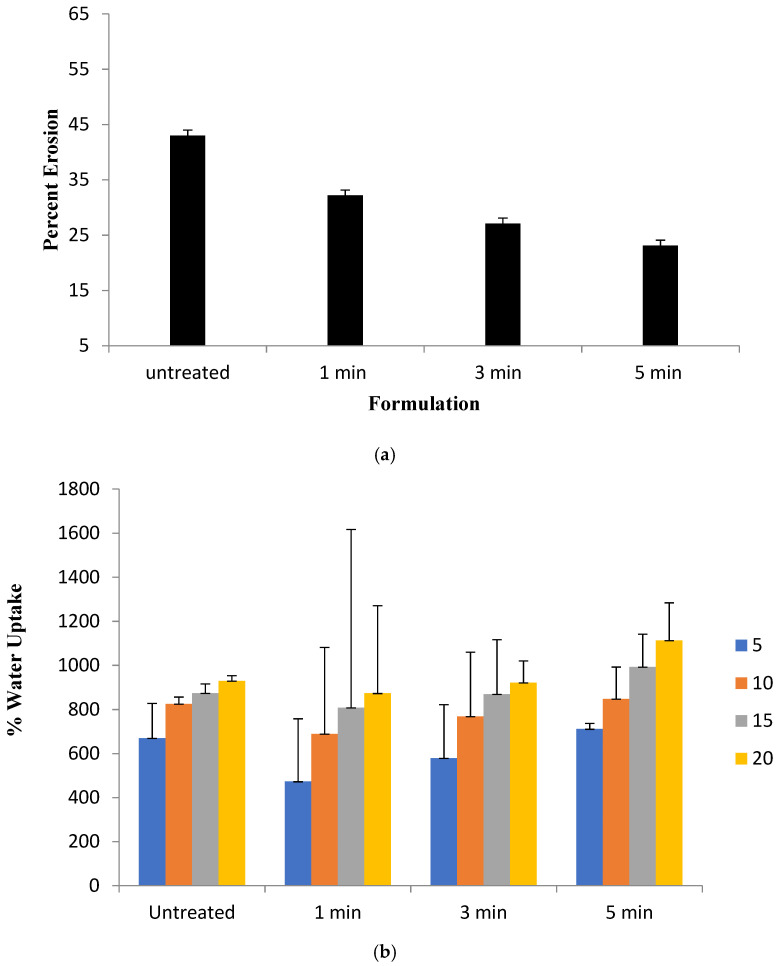
(**a**) Percent erosion; (**b**) water uptake of blend films.

The percent erosion and water uptake govern the fate of wound healing platform upon application to moist wounds. The percent erosion and water uptake results of untreated and microwave-treated film formulations are given in Figure 2. The untreated blend film eroded at a significantly faster rate (Student’s *t*-test, *p* < 0.05, Figure 2a) within 20 min of the immersion in phosphate buffer saline where it rapidly swelled and eroded because polymer hydrophilicity where chain relaxation proceeded quickly resulted in the loss of its structural integrity, and ultimately dissolution in the media [60], in comparison to microwave-treated blend film formulations where a significant reduction (Student’s *t*-test, *p* < 0.05, Figure 2a) in the ability of blend erosion was observed. The water uptake results of the untreated and microwave-treated blend films are given in Figure 2b. A slight, though insignificant (ANOVA, *p* > 0.05), rise in the water uptake capacity of the microwave-treated blend film for 5 min was observed in comparison to untreated as well as 1 min and 3 min treated film formulations.

### 3.4. Tensile Strength

The tensile strength results of the various film formulations are shown in Table 3. Microwave treatment resulted a gradual increase in the tensile strength in comparison to untreated formulation where a significant rise (Student’s *t*-test, *p* < 0.05) was observed for formulation with 5 min microwave treatment.

### 3.5. Vibrational Analysis

**Figure 3:**
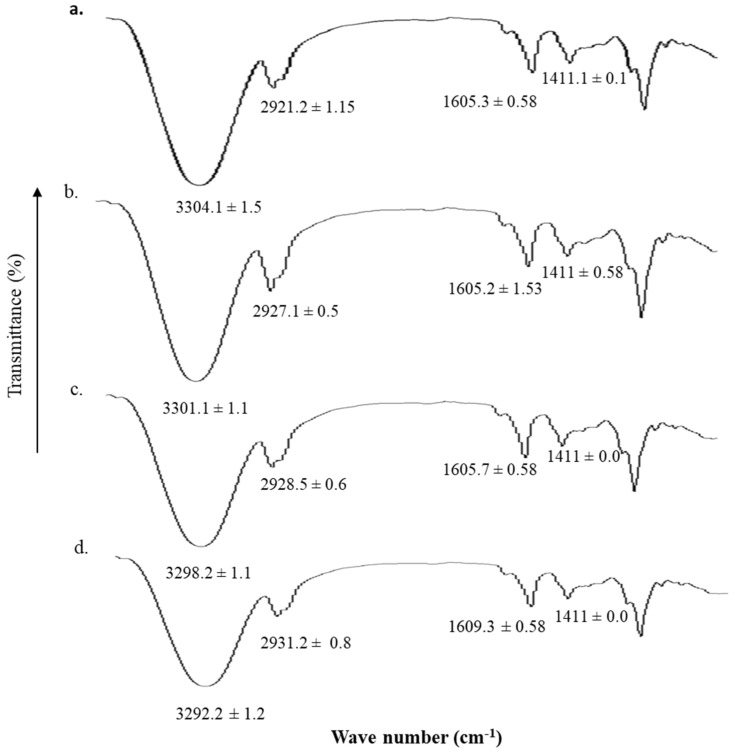
ATR-FTIR spectrum of (**a**) untreated, (**b**) 1 min, (**c**) 3 min, and (**d**) 5 min microwave treated pectin and sodium alginate blend film.

**Figure 4:**
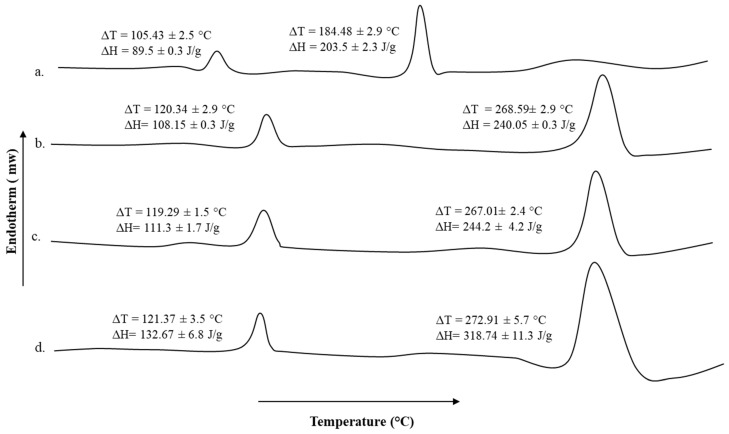
DSC thermogram of (**a**) untreated, (**b**) 1 min, (**c**) 3 min, and (**d**) 5 min microwave treated pectin and sodium alginate blend film.

The ATR-FTIR spectrum of untreated and blend films with different microwave time treatments are shown in Figure 3. The treatment of blend film with microwave for different time intervals resulted in significant changes in the corresponding wave numbers of hydrophilic as well as hydrophobic domains of the formulation. The hydrophilic OH/NH domain (3290–3305 cm^−1^) and C=O moiety (1604–1610 cm^−1^) underwent significant rigidification upon microwave treatment where 5 min treatment (Figure 4d) resulted in significant reduction (Student’s *t*-test, *p* < 0.05) in the corresponding wave number. Contrary to this, the hydrophobic asymmetric CH band (2920–2930 cm^−1^) undergoes significant fluidization with 5 min microwave treatment of the blend film where a significant rise (Student’s *t*-test, *p* < 0.05) in corresponding wave number from 2921.2 ± 1.15 cm^−1^ to 2931.2 ± 0.8 cm^−1^ was observed.

### 3.6. Thermal Analysis

The DSC thermogram of untreated sodium alginate-pectin blend and microwave treated blends for different time intervals are shown in Figure 4. The pectin moiety showed transition melting temperature of 105.43 ± 2.5 °C while the transition melting temperature of 184.48 ± 2.9 °C is ascribed to the sodium alginate moiety. The transition temperature and energy required to induce those transitions underwent significant rise for both pectin as well as sodium alginate moieties upon microwave treatments (Figure 4b–d). All microwave treatments significantly increased the transition temperatures of both polymer moieties as well as enthalpies in comparison to untreated blend formulation but a significant increase (Student’s *t*-test, *p* < 0.05, Figure 4d) in the enthalpies among various microwave treatments applied was observed with 5 min treatment, though the transition temperatures remain insignificant (Student’s *t*-test, *p* > 0.05, Figure 4d).

### 3.7. Scanning Electron Microscopy

**Figure 5:**
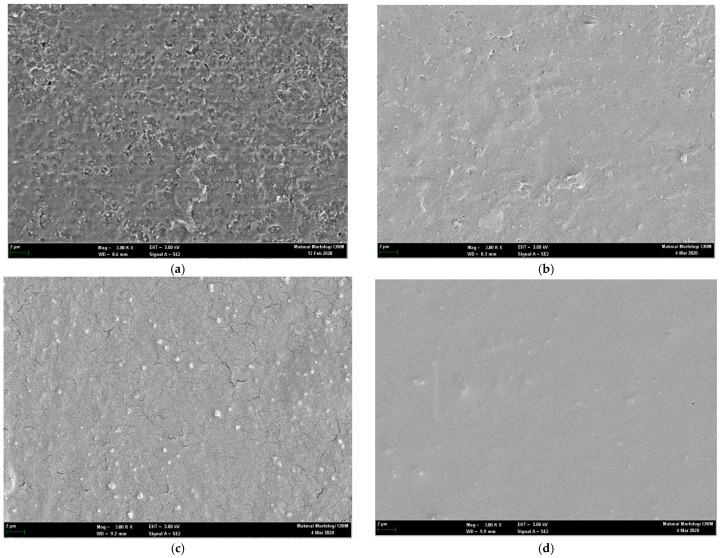
Scanning electron micrographs of (**a**) untreated, (**b**) 1 min, (**c**) 3 min, and (**d**) 5 min microwave treated pectin and sodium alginate blend film.

The surface morphological characteristics of untreated and microwave-treated blend films are shown in Figure 5. The untreated blend (Figure 5a) showed nonhomogeneous texture which could be due to migration of formulation constituents (tween 80 and/or glycerol) to the surface upon drying. The microwave treatment (Figure 5b–d) showed variable surface morphological results, where 1 min and 3 min microwave treatment showed surface adhesion of formulation constituents though to lesser extent than untreated one; formation of cracks (Figure 5c) depicting 3 min microwave treatment though facilitated polymer homogeneity but further drying process may have led to improper laying down of polymer fibers resulting in formation of spaces in the matrix. The 5 min microwave treatment (Figure 5d) resulted in more homogenous and uniform matrix deposition in a specific manner without any surface artifacts where even the further drying process did not render any destructive effect neither on the texture of the film nor on the formulation ingredients.

### 3.8. Wound Morphology

**Figure 6:**
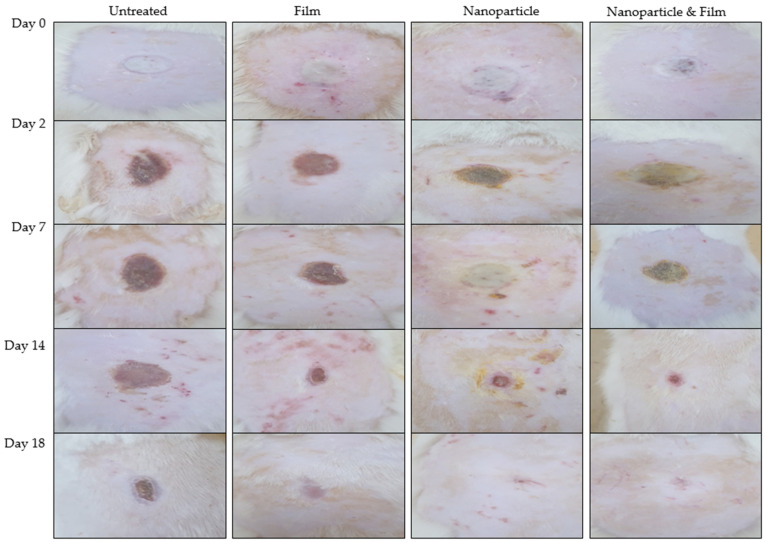
Wound images of rats with no applied scaffold (control) and treated by film, nanoparticles and combination of both on different days.

**Figure 7:**
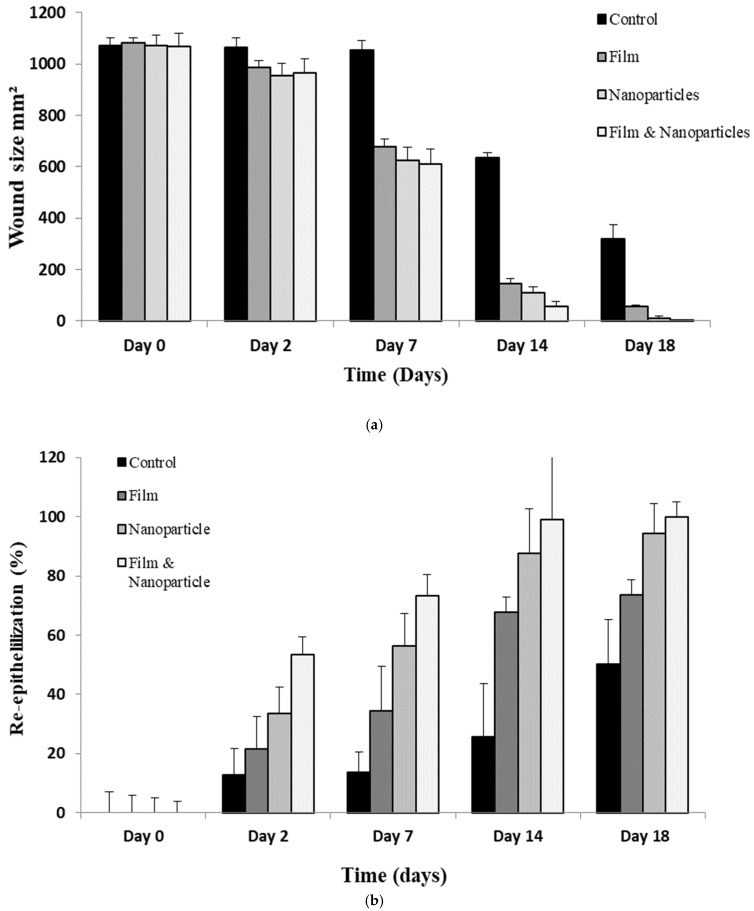
Profiles of (**a**) wound size and (**b**) state of re-epithelialization of rats.

The wound healing progress following 2nd degree burn wound infliction of various animal groups is shown in Figure 6. The untreated animal group depicted slow percent reepithelization and wound closure where the complete wound healing did not affect till day 18 as observed with test groups. Among test groups, the chitosan-curcumin nanoparticles alone as well as in combination showed significantly faster wound closure and percent reepithelization in comparison to untreated and only film treated animal group (ANOVA, *p* < 0.05, Figure 6) where complete wound closure as well as scare minimization was observed. Complete reepithelization took place within 18 days post treatment with nanoparticles and film combined application (Figure 7), while the untreated animals group showed complete wound closure over a time span of 28 days.

### 3.9. Vibrational Spectroscopic Analysis (ATR-FTIR) of Skin Samples

**Figure 8:**
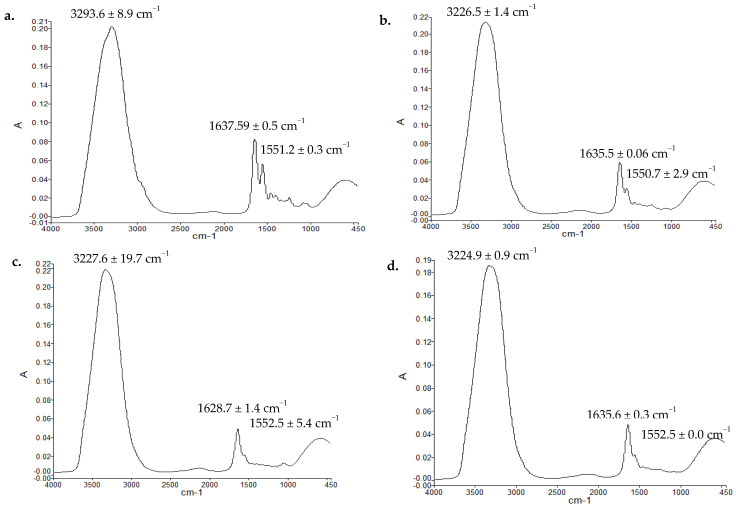
ATR-FTIR spectra of dermis of (**a**) control skin, skin treated by (**b**) film, (**c**) nanoparticles, (**d**) film and nanoparticle group.

The ATR-FTIR spectra of dermis layer of untreated and various test treated animal skin samples are given in Figure 8. Vibrational spectroscopic method of analysis of skin samples was used as a novel method of estimation of extent of proteins deposition as a function of applied treatments. For the purpose, OH/NH, amide-I and amide-II bands wave numbers and corresponding absorbance ratios with respect of untreated skin samples were recorded and calculated where the amide-I and amide-II as well as OH/NH bands typically originate from the protein moieties of the skin [61]. The changes in the wave numbers and absorbance ratios were assigned to increase and/or decrease in the rigidification of respective domains depicting higher population of corresponding protein moieties in the skin. The absorbance ratios are given in Table 4. The wave numbers of OH/NH domains (3222–3294 cm^−1^) in the untreated dermal layer significantly decreased (Students’ *t*-test, *p* < 0.05) with film, nanoparticles alone as well as nanoparticle-film combine application suggesting rigidification of hydrophilic OH/NH moieties of the dermal layer depicting formation of more compact skin structure. The absorbance ratio of OH/NH of test samples to untreated samples increased depicting more rigidification of hydrophilic domains of the dermal moieties (Table 4). Similarly, the wave number of amide-I (C=O stretching, 1627–1638 cm^−1^, Figure 8) moiety underwent significant decrease (Student’s *t*-test, *p* < 0.05) with various treatments applied while the amide-II moiety wave number (1549 to 1553 cm^−1^) did not show any significant changes (Students’ *t*-test, *p* > 0.05).

### 3.10. Thermal and Mechanical Strength Analysis of Skin Samples

**Figure 9:**
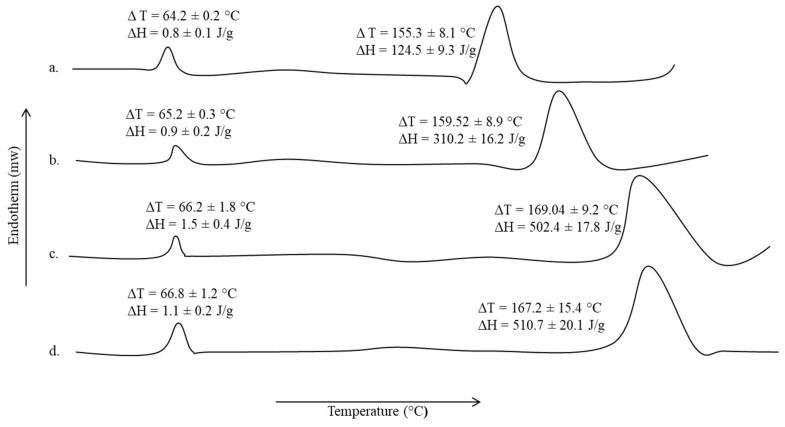
Thermograms of (**a**)untreated, (**b**) film treated, (**c**) nanoparticles treated, and (**d**) film-nanoparticles combined treated skin samples.

The DSC thermograms of test animal skin samples and untreated extracted from animals on 14 days of wounding are shown in Figure 9, while the tensile strength and percent elongation break are given in Table 5.

A slight increase in the transition temperature and corresponding enthalpy, though insignificant (Student’s *t*-test, *p* > 0.05) of lipidic domains (∆T = 64.02 ± 0.2 °C, ∆H = 0.8 ± 0.1 J/g, Figure 9) of the skin samples was observed with all treatments applied in comparison to untreated animal skin sample. However, the proteinous domain (∆T = 155.3 ± 8.1 °C, ∆H = 124.5 ± 9.3 J/g, Figure 9) underwent a significant rise not only in the corresponding transition temperature but also in the energy required to induce transition. The animal group treated with nanoparticles alone as well as in combination with film showed a significant rise in transition temperature and enthalpies (Student’s *t*-test, *p* < 0.05) in comparison to untreated and only film treated groups. The nanoparticle-film combined application hence translated into a significant rise in the tensile strength as well as percent elongation break (Student’s *t*-test, *p* < 0.05, Table 5) in comparison to untreated as well as other experimental animal groups.

### 3.11. Skin Histology

**Figure 10:**
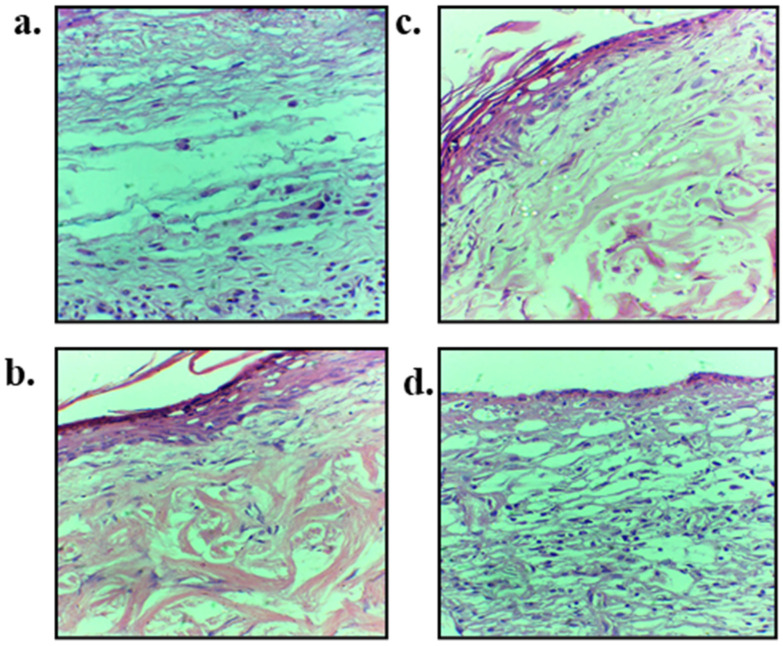
Hematoxylin and Eosin staining of skin samples extracted on day 14 of wounding on (**a**) untreated, (**b**) blend film, (**c**) nanoparticles and (**d**) nanoparticles-blend film combined treated skin samples.

**Figure 11:**
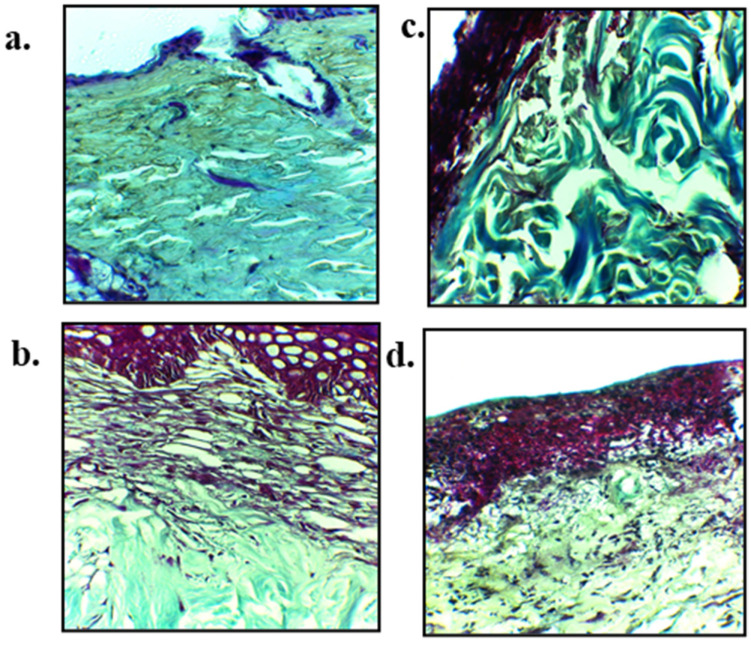
Masson trichrome staining of skin samples extracted on day 14 of wounding on (**a**) untreated, (**b**) blend film, (**c**) nanoparticles and (**d**) nanoparticles-blend film combined treated skin samples.

The hematoxylin and eosin (H&E) staining micrographs are shown in Figure 10 while Masson trichrome is shown in Figure 11. The H&E staining was performed on day 14 skin samples with an aim to elucidate the accumulation of inflammatory cells, regeneration of epidermis layer, blood vessels proliferation and tissue granulation formation. Control group showed poor epithelial regeneration along with sub-epithelial layer degeneration, and connective tissue fibers along with dermal glands were displaced in entire connective tissues (Figure 10a). Blend film treated skin exhibited partial epithelial regeneration followed by epidermal layer that regenerated over the granulation tissue and mild inflammatory infiltrate was noted (Figure 10b). Nanoparticles treated group with and without blend film application have connective tissue fibers that were properly arranged with many newly grown connective tissue, and entire dermal tissue in fiber bundles is visible. Connective tissue fibers were well developed and showed compactness in their structure without any displacement and sign of degeneration of tissue fibers (Figure 10d).

The emphasis of using Masson trichrome on burn wound tissue samples of untreated and various treated groups was to check the epidermal and dermal connective tissue fibers, as it gives green color to the collagen fibers which is very helpful in determining the extent of collagen production as well as its alignment at the healing area on 14 days post wounding (Figure 11). A significant difference in the extent and alignment of collagen fibers deposition following burn wound treated with nanoparticles (Figure 11c) and combine nanoparticles and blend film group (Figure 11d) was observed in comparison to untreated (Figure 11a) and film treated groups (Figure 11b), which demonstrated good synergistic wound healing characteristics of chitosan and curcumin. The untreated group showed void space in the dermis layer with irregular arrangement and disintegration of collagen fibers. Dermis and sub-epidermal regions also showed too much distortion followed by the epidermal region where epithelial integrity was distorted (Figure 11a).

## 4. Discussion

This project investigated the film and nanoparticles combined application as a novel strategy to treat burn wounds. As shown in Figure 1, the moisture adsorption ability of the blended films was directly related to microwave treatment time. Microwaves are electromagnetic waves which interact with polar moieties of the polymer in a volumetric manner [62]. Both polymers, i.e., sodium alginate and pectin, contain a number of polar functional groups viz OH/NH_2_, amide-I and amide-II, which may interact with each other upon microwave treatment preferably through hydrogen bonds [63]. Exposition of surface hydrophilic functional groups enables attraction and/or interaction with more water molecules [64]. The blend film also contained additional hydrophilic ingredients namely tween 80 and glycerol. The increase in the hydrophilicity of the blend film with microwave treatment might be due to arrangement of all hydrophilic components of the formulation towards the surface of the dried film matrix which enabled further increase in the attraction ability of water molecules towards the film surface. Furthermore, the polymer interlinking helps lay down the polymer fibers in a uniform fashion during drying process and results in formation of voids between polymer chains to form a controlled pore size throughout the polymer matrix [65]. Smaller pore size in the matrix translates into maximal retention and/or adsorption of moisture at the wound bed [66]. The pore size of the film is also considered important to minimize fibroblast/keratin in-growth into the film, thereby reducing secondary damage upon dressing changing [67]. The smaller pore size also enables reduction in the infiltration of opportunistic bacteria into wound bed, which translates into minimization of chances of secondary bacterial infections which may otherwise complicate the wound [68]. Furthermore, high surface adsorption capacity of film formulation also helps clear surface bacteria from the wound. The polymeric film enables bacterial adsorption possibly through biospecific/selective interaction (carbohydrate–protein, protein–protein) and nonspecific interactions (electrostatic or hydrophobic) [69] and hence film with high adsorption capacity may thus help in removing surface pathogenic bacteria from the wound bed [70]. The microwave based physically cross-linked sodium alginate and pectin blend film at frequency of 2450 MHz for 5 min is thus envisaged to promote skin tissue regeneration following 2nd degree burn by increasing the exudates removal from the wound bed due to controlled pore formation in the polymer matrix and hence prevent bacterial infiltration as well.

The reduced water loss from the wound bed into the external environment is of pivotal importance. Microwave treatment resulted in a significant increase in the *WVTR* and *WVP* (Table 2, Student’s *t*-test, *p* < 0.05). The *WVTR* and *WVP* are directly related to the pore size formed in the polymer matrix upon drying [71], while reduced *WVTR* and *WVP* are considered essential to prevent the wound from drying out. At the same time, appropriate pore size is also essential to facilitate gaseous exchange (oxygen and carbon dioxide) between the wound bed and environment [13]. A poor exchange of gases between the wound bed and external environment may accumulate CO_2_ leading to acidification of the wound media, which on one hand may directly inhibit the cell proliferation during angiogenesis and on the other hand may provide a favorable environment for the infiltration and growth of anaerobic bacteria [72]. In aerobic metabolism of glucose, generation of ATP’s are major energy molecules which drive the majority of the cellular processes during the wound healing process [73].Since healing tissue requires an increased energy demand [74], appropriate pore size of the polymer film, enough to facilitate gaseous exchange between wound bed and external environment, is thus envisaged to promote skin tissue regeneration.

The microwave treatment significantly delayed the erosion ability of the film (Student’s *t*-test, *p* < 0.05, Figure 2a), which could be attributed to crosslinking that restricts chain relaxation due to formation of egg box junctions among pectin and/or sodium alginate moieties, a time-dependent stabilization of the elicitor-active conformation which increases its biological activity [75]. Furthermore, reduction in the percent erosion with 5 min microwave treatment depicts formation of additional inter and intra polymer chains and/or polymer and other formulation moieties thus deterring the rapid entry of solvent molecules into the polymer matrix preventing rapid breakage of matrix structure [76]. Similarly, the water uptake capacity of the film determines the physicochemical properties of film like degradation, swelling, mechanical integrity, adhesivity, drug stability, drug release profile and biological response [77]. Reduced water uptake capacity determines the resistance of film to degradation in the wound physiological environment [78]. The water uptake ability of microwave-treated blend films (Figure 2b) demonstrated a slight increase in the water uptake capacity where the reason behind was not investigated but it is believed that microwave interaction with polar moieties of the polymers and other formulation ingredients may have resulted in the availability of more hydrophilic functional groups (OH/NH_2_, ester, amide) from the sodium alginate, pectin and/or tween 80 and glycerol resulting in a slight increase in the hydrophilic interaction with the available water. Although, high water uptake and slow erosion are deemed favorable to withstand shear and stress conditions during application as well as removal of exudate and debridement of the wound bed which translate into faster skin regeneration [13].

The tensile strength of the film formulation determines its fate following application as well as its ability to withstand shear stress during packaging and transportation; hence blend use is often advocated in place of lone polymer, which has higher mechanical strength [79,80]. Polymeric films that are intended to be used for wound healing applications should have appropriate mechanical properties that can support cellular activities such as proliferation, migration, and angiogenesis, as well as protect structures found in native skin such as blood vessels, lymphatic systems and nerve bundles [81]. For this purpose, the film should have mechanical properties similar to those found in native tissue. In this regard, the values of the tensile strengths (5 to 40 MPa), the Young’s modulus (4.5 to 25 MPa) and the percent elongation-to-break (35% to 120%) are considered appropriate for wound dressings [82] and sufficient to provide enough mechanical support for angiogenesis and tissue remodeling process during wound healing which also help in preventing stress shielding [83]. Microwave interacts with hydrophilic domains of the polymers (OH/NH_2_, amide-I, amide-II) [84], which are responsible for development of extra inter and intra polymer linkages preferably through H-bonding and/or electrostatic interactions [85]. Formation of additional linkages translates into stiffness of the polymer matrix thus formed and enables deposition of polymer fibers in a specific geometric manner during drying process [86]. The decrease in the spaces between the polymer fibers upon treatment results in more resistance to breakage and hence translates into higher mechanical stability [87]. This was the reason that 5 min microwave treatment significantly enhanced the mechanical stability of the blend film in comparison to untreated as well as other treatments.

The changes in hydrophilic and hydrophobic domains of the blend film (Figure 3) can be attributed to the interaction between the polymer chains and/or other formulation moieties namely tween 80 and/or glycerol which is envisaged to increase the mechanical strength as well as elasticity of the blends with microwave treatment in comparison to untreated. The interaction between the polar moieties of sodium alginate and pectin and/or polymers and tween 80/glycerol upon microwave treatment could have resulted in the formation of new bonds between them which resulted in the shrinkage of hydrophilic domains (OH/NH_2_, C=O, Figure 3b–d), which could aptly explain the blend film resistance to erosion and an insignificant rise in water uptake capacity due to reduction in the inter polymer chain spaces restricting rapid penetration of immersion medium molecules into polymer matrix. On the other hand, significant fluidization of hydrophobic moiety of the blend was observed which can aptly be attributed to the role of glycerol being a plasticizer in the blend film which resulted in a significant rise in the percent elongation as well as elastic modulus of the blend treated with microwave for 5 min [88]. The microwave activated polar functional groups and an increase in polar interactions between the polymers and/or other formulation entities translated into formation of new inter and intra molecular forces which might have enabled deposition of polymer chains in a layer-by-layer pattern to form a specific three-dimensional structure with controlled inter and intra polymer spaces [89].

Similarly, in thermal analysis (Figure 4) significant changes in the transition temperatures as well as enthalpies of the polymer moieties was observed with 5 min microwave treatment (Figure 4d). Since both pectin and sodium alginate have a number of hydrophilic functional groups with the ability to form inter and intra polymer H-bonding, this results in a rise in energy required to induce transition due to rise in propensity and extent of interaction between the various functional groups under microwave treatment [90]. Both pectin and sodium alginate are polysaccharide polymers and contain a number of OH and COOH functional groups which have the ability to form strong inter and intramolecular H-bonding [91]. Microwave interacts with polymers through polar functional groups in the polymer structure and elicit formation of additional linkages through H-bonding and/or electrostatic interaction [92]. The ability of microwave to develop a physically cross-linked structure resulted in a significant rise in the transition temperature as well as enthalpies of the blend which is envisaged to improve the physicochemical properties of the blend film.

The 5 min microwave treatment (Figure 5d) translated into a more homogenous and uniform matrix deposition in a specific manner without any surface artifacts where even the further drying process did not render any destructive effect, neither on the texture of the film nor on the formulation ingredients, suggesting 5 min microwave treatment of pectin and sodium alginate blend ensures proper amalgamation of all formulation ingredients which translates into smooth surface texture formation.

The modified chitosan-curcumin nanoparticles alone as well as in combination showed significantly faster wound closure and percent reepithelization in comparison to untreated and only film treated animal group (ANOVA, *p* < 0.05, Figure 6 and Figure 7). Curcumin is a natural wound healing compound with remarkable anti-inflammatory and collagen deposition properties [93]. Curcumin is reported to reduce expression of pro-inflammatory cytokines namely tumor necrosis factor alpha (TNF-α) and interleukin-1 (IL-1) [94]. Furthermore, curcumin has the inherent ability to recruit M2-like macrophages in the white adipose tissues which translate into enhanced production of anti-inflammatory cytokines which are detrimental for the inflammatory response [95] and also inhibit nuclear factor (NF-kB), thus reducing inflammation at the wound site [96]. Curcumin is also reported to recruit wound site fibroblasts in vitro and in vivo by inciting proliferation and migration which is partially mediated by Dkk-1, where curcumin has been speculated to precisely control the Dkk-1 level in a temporospatial manner to regulate the downstream Wnt signaling pathway which is necessary to facilitate fibroblasts proliferation and migration at wound site [97]. The wounds are also associated with formation of reactive oxygen species (ROS) particularly hydrogen peroxide (H_2_O_2_) and superoxide (O_2_^−^) which are associated with prolonging the inflammatory phase of the wound healing due to damage caused to healing cells [98]. Curcumin acts as a potent antioxidant by scavenging ROS and suppression of transcription factors related to oxidation, which is conferred by its electron donating functional groups (i.e., the phenolic OH groups) [99].

Chitosan is a promising polymer with anti-bacterial [100], anti-inflammatory [101] and hemostatic properties [102]. The antimicrobial properties of the chitosan are due to its positive surface charge due to protonation of NH_2_ functional groups which interact with negatively charged microbial cell membrane and/or cell wall and thus incite lysis [103]. The extent of positive surface charge on chitosan is directly related to its degree of deacetylation [104]. The chitosan variant used had a degree of deacetylation 91.41 ± 8.2, thus increased propensity of positive surface charge was imparted to nanoparticles. The chitosan-curcumin nanoparticles are thus envisaged to exert synergistic antimicrobial activity. Furthermore, chitosan has also been reported to facilitate wound healing by promoting growth of macrophages and fibroblasts where secretion of cytokines such as transforming growth factor-β (TGF-β), PDGF and IL-1 which translate into migration of macrophages into wound site, promoting fibroblasts proliferation and enhancing collagen secretion [105]. This could be the reason that chitosan-curcumin nanoparticles hasten percent reepithelization process and wound closure in comparison to untreated and film animal groups. The percent re-epithelization was significantly higher when nanoparticles and film were used in combination (Figure 7). The nanoparticle and film combined application facilitated rapid wound healing by preventing rapid water loss from the wound bed and facilitated easy gaseous exchange between the wound bed and external environment which translated into rapid wound closure and higher percent of reepithelization.

The vibrational spectroscopic analysis of skin samples and absorbance ratios of various absorption bands excised at specific time interval (Figure 8, Table 4) underwent significant changes in the propensity of skin proteinous domains. The spectral bands in vibrational analysis of samples are molecule-specific and are directly associated with the vibrations of particular chemical bonds and/or functional groups when exposed to IR radiations [106], while the absorbance values are directly related to the propensity and/or concentration of those particular molecules where higher absorbance values indicate higher intensity of that specific region [107]. The higher absorbance ratios observed with nanoparticles-film combined application clearly indicate that more proteins (predominantly collagen) are being deposited at the wound site as compared to other treatments applied [108].

As shown in Figure 9, nanoparticles application alone as well as in combination with film translated into a significant rise in the transition temperature as well as enthalpies of the proteinous domains of the skin samples as well as a significant rise in the mechanical strength of the skin (Table 5). Curcumin is found to increase collagen synthesis, cellular proliferation and hence increase collagen deposition for rapid wound healing [98].Chitosan, with high degree of deacetylation, promotes wound healing and increases fibroblast activity and uniform collagen deposition translating into higher tensile strength [109]. The increased transition temperature and corresponding enthalpy observed with nanoparticles treated group and nanoparticles-film combined treated group is thus the synergistic activity of curcumin as well as chitosan to promote skin tissue regeneration, while the film provided an additional support to keep the wound moist which is detrimental for rapid wound closure. The nanoparticle-film combined application hence translated into a significant rise in the tensile strength as well as percent elongation break (Student’s *t*-test, *p* < 0.05, Table 5) in comparison to untreated as well as other experimental animal groups, which is envisaged to be due to synergistic action of curcumin and nanoparticles in ensuring rapid formation of collagen fibers and their uniform deposition at wound site.

The hematoxylin and eosin (H&E) staining micrographs (Figure 10) showed application of nanoparticles alone as well as in combination with film resulted in formation of properly arranged connective tissue fibers. Chitosan has been reported to possess antimicrobial [110], antioxidant [111] and immunomodulatory activity [112]. The moist environment of the wound post skin injury provides a favorable environment for the microbes to infiltrate the wound and hence induce secondary bacterial infection [113]. The antimicrobial activity of chitosan is directly linked to its degree of deacetylation as chitosan variant having a higher number of free NH_2_ groups reported to possess higher antimicrobial activity due to their enhanced ability to interact electrostatically with the negatively charged microbial cell membrane [114]. Similarly, the antioxidant activity of the chitosan is also directly linked to its molecular weight and degree of deacetylation where low molecular weight and higher degree of deacetylation are reported to exert higher free radical scavenging ability [115]. Chitosan possesses the ability to inhibit proinflammatory cytokines and promote tissue granulation through fibroblast recruitment [116]. Friedman et al. investigated the P. *acnes* induced cytokines and chemokines and found a significant reduction in levels of IL-12p40 demonstrating complete reduction of IL-12 and IL-6 [117]. On the other hand, curcumin is a natural compound which possesses remarkable antioxidant, antimicrobial, and anti-inflammatory properties [118]. The anti-inflammatory activity of curcumin was reported to be through peroxisome proliferator-activated receptor gamma (PPAR-γ) [119]. Furthermore, curcumin also exerts its anti-inflammatory activity through T cells, B cells, neutrophils, natural killer cells, dendritic cells, and macrophages [120]. The synergistic anti-inflammatory activity of chitosan and curcumin is thus envisaged to promote rapid wound closure and formation of denser and more well-developed connective fibers and higher fibroblasts accumulation at wound site independent of film application (Figure 11d).

Similarly, a synergistic activity of chitosan and curcumin was observed when nanoparticles were applied in combination with film in the alignment at the healing area 14 days post wounding (Figure 11). Chitosan possesses the ability to regulate granulation tissue formation and promote angiogenesis, thus assuring correct deposition of collagen fibers which translate into correct repair of injured dermal tissue [121]; while curcumin also promotes collagen deposition by enhancing the fibroblast proliferation, vascular density and modulating the granulation tissue formation which facilitate extracellular matrix production at the wound site [122]. It also induces transforming growth factor-β and thus stimulates angiogenesis and accumulation of extracellular matrix continues through the remodeling phase of wound healing [123]. The synergistic collagen deposition ability of chitosan and curcumin translated into more compact collagen fiber deposition in case of nanoparticles alone as well as when applied in combination with blend film in comparison to untreated and film treated animal group.

## 5. Conclusions

This study explored the combinational use of modified chitosan-curcumin nanoparticles and microwave enabled physically cross-linked polymer blend film for skin regeneration potential following 2nd degree burn wound in animals. The microwave treatment significantly improved the physicochemical attributes of polymer blend film addressing the properties required of a wound healing platform. The cross linking between the two polymers was initiated by rigidification of hydrophilic and fluidization of hydrophobic domains which resulted in a significant change in the corresponding transition temperature as well as enthalpies of the polymer moieties. The combinational application of modified chitosan-curcumin nanoparticles hastens the skin regeneration process and translates into a significant increase in the extent of protein deposition as well as mechanical strength. The combined use of microwave enabled physically cross-linked sodium alginate and pectin film and modified chitosan-curcumin nanoparticles may open new horizons in treatment strategies of burn wounds.

## Figures and Tables

**Table 1:** Different formulations of sodium alginate pectin blend film that were microwave treated.

Formulation	Sodium Alginate (*w/w*)	Pectin (*w/w*)	Glycerol (2%, *w/w*)	Tween-80 (0.1%*w/w*)	Distilled Water (*w/w*)	Microwave Treatment Time (Min)
Untreated	2 g	2 g	2 g	0.1 g	93.9 g	0
1 min	2 g	2 g	2 g	0.1 g	93.9 g	1
3 min	2 g	2 g	2 g	0.1 g	93.9 g	3
5 min	2 g	2 g	2 g	0.1 g	93.9 g	5

**Table 2:** *WVTR* and *WVP* results of different formulations.

Formulation	Film Thickness (mm)	WVTR (g/m^2^h)	WVP (g mm/h·m^2^)
Untreated blend	0.22 ± 0.03	180 ± 1.8	0.152 ± 0.06
1 min treated blend	0.18 ± 0.02	240 ± 0.6	0.164 ± 0.02
3 min treated blend	0.19 ± 0.03	256 ± 0.6	0.177 ± 0.03
5 min treated blend	0.20 ± 0.03	276 ± 0.5	0.191 ± 0.02

**Table 3:** Mechanical properties of various film formulations.

Formulation	Tensile Strength (Mpa)	Elongation Break (%)	Elastic Modulus (Mpa)
Untreated blend	2.34 ± 0.24	40.02 ± 5.52	9.39 ± 12.50
1 min treated blend	5.21 ± 0.41	43.34 ± 2.98	60.28 ± 15.55
3 min treated blend	7.88 ± 0.04	50.01 ± 4.71	79.99 ± 25.98
5 min treated blend	9.94 ± 0.08	66.66 ± 3.29	86.78 ± 22.98

**Table 4:** Absorbance ratios of various regions.

Samples	Absorbance Ratios of OH/NH of Test to Untreated Dermal Moieties	Absorbance Ratios of Amide-I (C=O Stretching) to Amide-II (C-N Bending) of Various Test Dermal Moieties
Film	0.91 ± 0.005	1.01 ± 0.05
Nanoparticle	1.05 ± 0.005	1.8 ± 0.01
Nanoparticle-film combined	1.12 ± 0.015	2.15 ± 0.001

**Table 5:** Mechanical properties of skin sample.

Animal Groups	Tensile Strength (MPa)	Elongation Break (%)	Elastic Modulus (MPa)
Untreated	8.61 ± 0.7	12.09 ± 0.7	1.82 ± 2.1
Film	10.98 ± 0.6	14.89 ± 0.5	3.32 ± 2.9
Nanoparticles	13.78 ± 0.9	17.14 ± 1.5	4.10 ± 1.9
Nanoparticles-film combined	14.65 ± 0.8	17.99 ± 0.7	5.78 ± 1.7
